# Longitudinal Study of the Role of Epidermal Growth Factor on the Fractional Excretion of Magnesium in Children: Effect of Calcineurin Inhibitors

**DOI:** 10.3390/nu10060677

**Published:** 2018-05-27

**Authors:** Kristien J. Ledeganck, Charlotte Anné, Amandine De Monie, Sarang Meybosch, Gert A. Verpooten, Marleen Vinckx, Koen Van Hoeck, Annelies Van Eyck, Benedicte Y. De Winter, Dominique Trouet

**Affiliations:** 1 Laboratory of Experimental Medicine and Pediatrics, University of Antwerp, Universiteitsplein 1, T3.34, 2610 Antwerp, Belgium; charlotte.anne@student.uantwerpen.be (C.A.); amandine.demonie@student.uantwerpen.be (A.D.M.); sarang.meybosch@student.uantwerpen.be (S.M.); gert.verpooten@uantwerpen.be (G.A.V.); marleen.vinckx@uantwerpen.be (M.V.); Koen.vanhoeck@uza.be (K.V.H.); annelies.vaneyck@uantwerpen.be (A.V.E.); benedicte.dewinter@uantwerpen.be (B.Y.D.W); Dominique.trouet@uza.be (D.T.); 2 Department of Pediatric Nephrology, Antwerp University Hospital, Wilrijkstraat 10, 2650 Edegem, Belgium

**Keywords:** epidermal growth factor, magnesium, children, magnesium intake, fractional excretion, calcineurin inhibitor, kidney transplantation, nephrotic syndrome

## Abstract

Background: It was shown in animal models and adults that the epidermal growth factor (EGF) is involved in the pathophysiology of calcineurin inhibitor (CNI) induced renal magnesium loss. In children, however, the exact mechanism remains unclear, which was set as the purpose of the present study. Methods: Children with nephrotic syndrome and renal transplant children treated with CNI (*n* = 50) and non-CNI treated children (*n* = 46) were included in this study. Urine and serum samples were collected at three time points to determine magnesium, creatinine, and EGF. The magnesium intake was calculated from a food frequency questionnaire. Results: Serum Mg^2+^ and urinary EGF/creatinine were significantly lower in the CNI treated children, with significantly more CNI-treated children developing hypomagnesaemia. In the latter patients, the fractional excretion of magnesium (FE Mg^2+^) was significantly higher. Urinary EGF, age, renal function, and serum magnesium were independent predictors of the FE Mg^2+^. Only 29% of the children reached the recommended daily intake of magnesium. The magnesium intake did not differ between hypomagnesemic and normomagnesemic patients and was not a predictor of the FE Mg^2+^. Conclusions: In CNI-treated children who developed hypomagnesemia, the FE Mg^2+^ was increased. The urinary EGF concentration, age, and renal function are independent predictors of the FE Mg^2+^.

## 1. Introduction

Magnesium (Mg^2+^) is an intracellular cation with roles in multiple physiologic processes, such as parathyroid metabolism, cardiovascular tone, nerve conduction, and the proper function of adenosine triphosphate complexes [1]. Furthermore, it also plays an important role in bone metabolism [2]. In the growing child, it is, therefore, essential to maintain a positive Mg^2+^ balance so that the amount of Mg^2+^ needed for growth and metabolic needs is ensured [2]. This balance requires interaction between the gut, the kidney, and the bone. Mg^2+^ is absorbed from the food in the gut and stored in the bone, while Mg^2+^ excess is excreted by the kidneys in the feces [3].

The recommended Mg^2+^ intake in children depends on their age, with increasing needs during adolescence to meet pubertal growth needs. The recommended intake approximates 130–150 mg/day for ages 4–8 year [2], 240 mg/day for ages 9–13 year, and 340 mg/day for boys and 300 mg/day for girls aged 14–18 year [4]. Magnesium deficiency is rare in healthy subjects as Mg^2+^ is widely present in food sources, such as dairy, nuts, whole cereal grains, green vegetables, dark chocolate, and legumes [5,6,7]. However, in a Cypriot population, none of the children aged 6–19 year met the recommended Mg^2+^ intake, with the highest prevalence of insufficiency in the adolescents [8]. Additionally, in chronic kidney disease (CKD) patients, magnesium intake has been shown to be below the recommended level [9]. The main nutrition-related goals for CKD patients involve slowing of the kidney failure progression rate, maintaining good nutritional status, and minimizing CKD complications, such as metabolic disorders and proteinuria. The nutrition requirements differ among patients with various stages of kidney function and proper nutrition is, therefore, difficult to fulfil in CKD patients [10]. Hypomagnesemia is defined as a serum Mg^2+^ concentration below 0.7 mmoL/L [11]. Most cases of hypomagnesemia in clinical practice, however, are asymptomatic. The clinical manifestation may depend more on the total body Mg^2+^ deficit rather than on the actual serum Mg^2+^ levels. Personality changes, muscle weakness, tremor, and dysphagia may occur at concentrations of <1.45 mg/dL, while confusion and a decreased consciousness develop at concentrations of ≤1.00 mg/dL [12]. Hypomagnesemia has been reported in children with malignancy and in those being treated for malnutrition [1]. In hospitalized children at the pediatric intensive care unit, a 20% to 60% incidence of hypomagnesemia has been reported [1].

Calcineurin inhibitors (CNIs), especially cyclosporine-A (CsA) and tacrolimus (TAC), are widely used immunosuppressive agents. Since the 1970s, CNIs are commonly administered to kidney transplant recipients or after other solid organ transplantation to reduce the rejection rate and improve early graft survival, although long-term nephrotoxicity is a serious side effect [13]. CNIs are also used for the treatment of steroid-resistant or steroid-dependent idiopathic nephrotic syndrome [13].

Besides nephrotoxicity, CNIs also induce functional alterations and ion homeostasis disturbances, such as hypomagnesemia and renal magnesium wasting, hyponatremia, hyperkalemia, hyperchloremic metabolic acidosis, and hyperuricemia [14,15]. Two ion channels play an important role in the Mg^2+^ homeostasis, TRPM6 and TRPM7. TRPM6 has an expression pattern predominantly present in absorbing epithelia. In the kidney, TRPM6 is expressed in the distal convoluted tubule, known as the main site of active transcellular Mg^2+^ reabsorption along the nephron. TRPM7 is ubiquitously expressed and implicated in cellular Mg^2+^ homeostasis, cell death, and cell cycle regulation [16,17]. A decade ago, it was demonstrated that epidermal growth factor (EGF) plays a crucial role in the stimulation of the renal Mg^2+^ channel TRPM6 [18,19]. Our research group demonstrated in a rat model that a combined decrease in the expression of renal EGF and the Mg^2+^ channel TRPM6 is responsible for CsA-induced renal Mg^2+^ loss [20]. In a translational clinical setting in adults, an increased FE Mg^2+^ was observed in CsA-treated patients who developed hypomagnesemia associated with decreased urinary renal EGF levels [21].

Besides its role in magnesium homeostasis, EGF is involved in many biological responses, including cell proliferation, differentiation, and migration, as well as pathophysiological events, such as tissue repair including ulcer and wound healing, or tissue repair, after ischemia/reperfusion injury [22,23]. EGF is also involved in inflammation, showing protective effects in animal models of pancreatitis [24]. Moreover, the correlation between EGF and other growth factors, such as transforming growth factor β (TGF-β) and platelet derived growth factor, needs to be considered [25,26].

In the present study, we aimed to investigate if children who were treated with CNIs developed hypomagnesemia and/or renal Mg^2+^ loss, and if the urinary EGF expression level is related to the fractional excretion of Mg^2+^ (FE Mg^2+^). Furthermore, we investigated whether Mg^2+^ intake was related to the serum Mg^2+^ level and the FE Mg^2+^ in patients treated with CNIs.

## 2. Materials and Methods 

### 2.1. Study Design

We performed a longitudinal observational clinical trial. Patients were included between March 2016 and February 2018 at the Antwerp University Hospital. Ninety-six patients were recruited in this study and divided into 2 subgroups. Group 1: Patients treated with CNI (*n* = 50); subdivided into renal transplant patients treated with CNI (*n* = 23; group 1A) and patients with nephrotic syndrome treated with CNI (*n* = 27; group 1B). Group 2: Non-CNI treated patients (*n* = 46); subdivided into patients with CKD as a control group for group 1A (*n* = 24; group 2A) and patients with nephrotic syndrome not treated with CNI as a control group for group 1B (*n* = 22; group 2B). Exclusion criteria were an estimated glomerular filtration rate (eGFR) < 20 mL/min/1.73 m^2^, use of cisplatin, diuretics or aminoglycosides, diabetes mellitus, and an active urinary tract infection.

At 3 time points, with an interval of at least 1 month, blood and urine samples were collected from each patient to determine creatinine, magnesium, and EGF (urine). In the nephrotic syndrome patients, samples were collected during a period of remission. At one time point during the study, a Food Frequency Questionnaire was performed to obtain the frequency and portion size information. 

A healthy control group (*n* = 103 healthy children) was included to determine serum and urine EGF reference values in children. From these patients, age, gender, weight, and length were obtained. From 42 patients, at 1 time point, both urine and serum were sampled, from 31 patients, only urine was sampled, and from 30 patients, only serum was sampled. 

The study was conducted in accordance with the *Declaration of Helsinki* and the principles of Good Clinical Practice. The study protocol was approved by the Ethics Committee of the Antwerp University Hospital (file number 9/44/231). All patients and their parents and/or legal guardians gave a written informed consent.

### 2.2. Magnesium Intake Questionnaire and Nubel^®^

A Food Frequency Questionnaire was performed to obtain standardized information on food intake, with special attention to the frequency and portion size information. This information was then entered into the dietary software program Nubel^®^ food planner (Nubel v.z.w. Eurostation, Brussels, Belgium) to analyse the daily Mg^2+^ intake. Nubel^®^ is a Belgian non-profit organization responsible for the management of the nutritional and scientific information of food products in Belgium [27]. In patients treated with magnesium supplements, the amount of magnesium substitution was added to the daily magnesium intake from the food to calculate the total magnesium intake. The magnesium intake was then calculated as a percentage of the reference daily intake (RDI; Nubel) and thus corrected for age.

### 2.3. Determination of Creatinine, Magnesium, and CsA Levels

Serum and urine creatinine and magnesium, and whole blood CsA levels were analyzed with the Dimension Vista system (Siemens Healthcare Diagnostics, Deerfield, MA, USA) using an ECREA, Mg, or CsA flex^®^ reagent cartridge, respectively. FE Mg^2+^ was calculated using the following equation: FE_Mg_ = 100 × (U_Mg_ × S_Cr_)/[(0.8 × S_Mg_) × U_Cr_], with U_Mg_ urinary excretion of Mg^2+^ (mg/dL), S_Cr_ serum creatinine (mg/dL), S_Mg_ serum Mg^2+^ (mg/dL), and U_Cr_ urinary excretion of creatinine (mg/dL). The serum Mg^2+^ concentration was multiplied by 0.8, since, in children, only 80% of the serum Mg^2+^ is freely filtered by the glomerulus, with the remaining part being protein-bound [28].

Creatinine clearance was calculated using the Bedside Swartz equation, which is the recommended equation to estimate the GFR in children [29].

Since it is known that estradiol might influence renal magnesium reabsorption [30], serum estradiol levels were measured in female children using the Elecsys Estradiol III Assay (Cobas^®^, Roche Diagnostics GmbH, Mannheim, Germany). TAC levels were measured with the Elecsys Tacrolimus Assay.

### 2.4. Determination of Urinary EGF

Urinary EGF was measured using an EGF human Elisa kit^®^ (Invitrogen, Waltham, MA, USA), according to the manufacturer’s guidelines. The detection limit of this assay was 3.9 pg/mL. A preliminary experiment (*n* = 10) was performed to test the intra- and inter-variability of the EGF human Elisa kit^®^, showing a mean intra-assay coefficient of variance of 9.82% and an inter-assay coefficient of variation of 9.81%.

### 2.5. Statistical Analysis

All data were analysed using SPSS (version 24.0). Statistical significance was predetermined as *p*-value < 0.05. Normality of cross-sectional data (such as magnesium intake) was tested by applying the Kolmogorov-Smirnov test. Parametric data are expressed as mean ± standard deviation (SD) and non-parametric data are expressed as median (minimum-maximum). The correlation of magnesium intake with other variables was tested with a Pearson or Spearman correlation test for parametric or skewed data, respectively. Generalized Estimating Equations (GEE) were used to analyse the data collected per visit since the groups had unequal sample sizes. Moreover, the number of visits per patient varied from 1 to 3. GEEs were used to calculate the estimated means of variables and to compare them between groups. Models were constructed to study the time-dependency of the variables and to determine the relationship between outcome variables, such as FE Mg^2+^ and urinary EGF excretion, and a number of other predictors.

## 3. Results

### 3.1. Population Demographics

#### Group Descriptions

From 89% of the patients, three consecutive samples were collected, while from 9% of the patients only two samples were collected, and from 2% of the patients only one sample was collected. In the entire study population, the median age was 12.0 year (2.3–20.3 year). Seventy-one percent of the patients were male, the mean weight was 43.0 ± 18.0 kg, the median length was 147.5 cm (88–190 cm), and the median BMI *z*-score was 0.16 (−2.24–2.82).

The demographic data per group are displayed in Table 1.

### 3.2. Kidney Function, Magnesium, EGF, and CNI Levels

In the entire study population, the median serum creatinine was 0.77 mg/dL (0.27–2.97 mg/dL), the creatinine clearance was 80 mL/min/1.73 m^2^ (19–180 mL/min/1.73 m^2^), serum Mg^2+^ was 0.80 mg/dL (0.42–1.22 mg/dL), and the FE Mg^2+^ was 4.70% (0.16–21.47%). From all the patients, 29.3% developed hypomagnesemia at least at 1 time point during the study. The mean urinary EGF was 10.51 ng/mL (1.14–182.70 ng/mL) and the mean serum EGF was 811.73 pg/mL (33.07–2014.33 pg/mL). A detailed overview per group is shown in Table 2.

In the female patients (*n* = 28), the median estrogen level was 34.86 pg/mL (2.5–268.3 pg/mL) and did not differ between groups. From these patients, 29.6% had serum estrogen levels below the detection limit.

#### Patients with Hypomagnesemia versus Patients with Normomagnesemia (presented in Table 3)

Twenty-nine percent of the patients developed hypomagnesemia. Patients who developed hypomagnesemia showed a higher FE Mg^2+^ when treated with CNI. The FE Mg^2+^ was 10.4% (1.8%) in the renal Tx group and 5.9% (1.7%) in the CKD group (*p* = 0.073), and 4.9% (0.6%) in the nephrotic syndrome group treated with CNI compared to 3.5% (<0.1%) in the nephrotic syndrome group not treated with CNI (*p* = 0.023). There was no difference in magnesium intake between hypomagnesemic patients treated with CNI and both control groups (*p* = 0.243).

### 3.3. Magnesium Intake

The median amount of magnesium intake was 87% (37–684%) of the reference daily intake (RDI, corrected for age). Only 29% of all patients exceeded the RDI. Although the median magnesium intake remained stable from 8 year on, the number of children that reached the RDI for magnesium intake was decreasing with age: 63.2% of the children < 8 year exceeded the RDI for magnesium intake (median amount was 119% (62–684%)), while this was only 17.9% of children between 8–12 year (77% (41–151%)), 33.3% between 12–16 year (80% (56–436%), and 21.1% of the >16 year old children and adolescents (86% (37–323%)).

**Figure 1:**
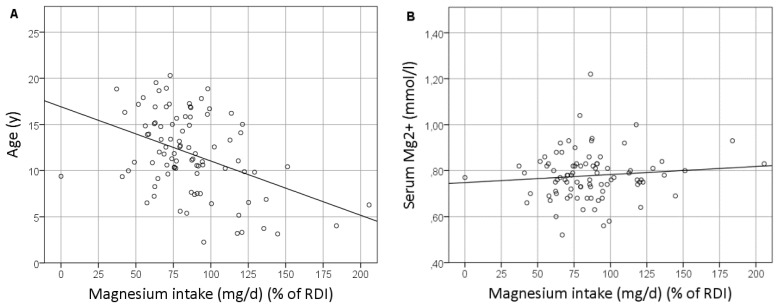
Correlation between the magnesium intake and age (**A**) and the serum magnesium level (**B**). RDI: Recommended daily intake.

Magnesium intake was significantly correlated with age (Figure 1A; *p* < 0.001; *r* = −0.375), weight (*p* = 0.009; *r* = −0.272), and length (*p* = 0.002; *r* = −0.318). The use of Mg^2+^ supplements was negatively correlated with magnesium intake (*p* = 0.095; *r* = −0.177). Magnesium intake did not correlate with other variables, such as the BMI *z*-score, sex, serum Mg levels (Figure 1B), renal function, serum or urinary EGF concentration, or the presence of hypomagnesemia.

### 3.4. The Healthy Control Group

The median age in the healthy control group was 13.2 year (3.3–17.9 year). Fifty-four percent of the healthy control children were male and length, weight, and BMI *z*-score was comparable to the study groups.

The serum creatinine was 0.54 mg/dL (0.32–1.09 mg/dL), the creatinine clearance was 108 mL/min/1.73 m^2^ (66–143 mL/min/1.73 m^2^), serum Mg^2+^ was 0.92 mg/dL (0.80–1.07 mg/dL), and the FE Mg^2+^ was 3.45% (0.81–5.82%). None of the patients developed hypomagnesemia.

The median urinary EGF was 67.4 ng/mL (17.9–218.8 ng/mL) and the median serum EGF was 807.7 pg/mL (141.6–2087.0 pg/mL). The urinary and serum EGF strongly correlated with age (*p* < 0.001; *r* = −0.406 for urinary EGF and *r* = −0.447 for serum EGF). Urinary EGF also correlated with creatinine clearance (*p* = 0.011; *r* = 0.408), but not with sex (*p* = 0.514; *r* = −0.079), FE Mg^2+^ (*p* = 0.819; *r* = 0.038), serum Mg^2+^ (*p* = 0.478; *r* = 0.119), or serum EGF (*p* = 0.405; *r* = 0.135). Serum EGF also correlated with the BMI *z*-score (*p* = 0.001; *r* = 0.369) but not with sex (*p* = 0.054; *r* = 0.231), creatinine clearance (*p* = 0.520; *r* = 0.108), serum Mg^2+^ (*p* = 0.478; *r* = 0.119), or FE Mg^2+^ (*p* = 0.231; *r* = −0.199).

### 3.5. Predictors of FE Mg^2+^

**Figure 2:**
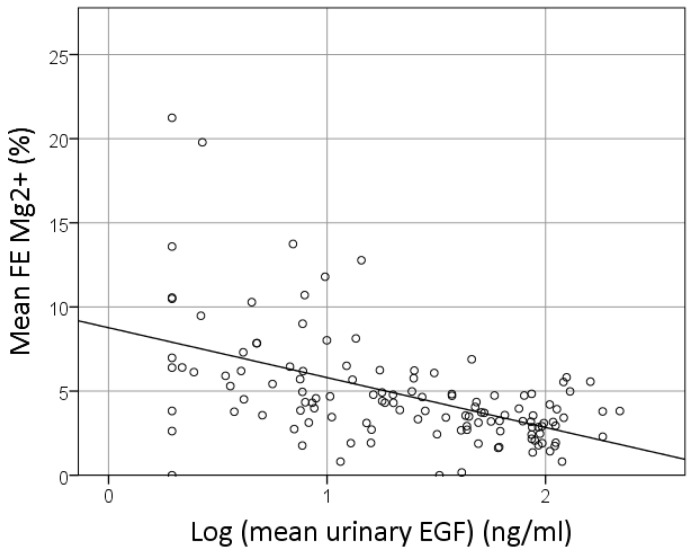
Correlation between the EGF and fractional excretion (FE) of magnesium. Logarithmic normalized data of the EGF are presented.

In a univariate GEE analysis, the FE Mg^2+^ was predicted by the urinary EGF concentration, with *r* = −0.57 and *p* < 0.001 (depicted in Figure 2).

Urinary EGF, age, sex, renal function, serum magnesium concentration, the urinary protein/creatinine ratio, and Mg^2+^ intake were tested in a multivariate GEE model as predictors of FE Mg^2+^. Except for sex (*p* = 0.747), urinary protein/creatinine ratio (*p* = 0.192), and Mg^2+^ intake (*p* = 0.593), the other factors appeared to be independent predictors of FE Mg^2+^ (data presented in Table 4).

## 4. Discussion

This clinical study revealed several new insights into magnesium homeostasis in children: (1) Children treated with calcineurin inhibitors had lower serum magnesium levels and, more frequently, developed hypomagnesemia; (2) In children who developed hypomagnesemia, the FE Mg^2+^ was increased in patients treated with calcineurin inhibitors; (3) The urinary EGF concentration and the FE Mg^2+^ were inversely correlated in all research groups; (4) We defined a predictive model for the FE Mg^2+^ in children, including the urinary EGF concentration, age, renal function, and the serum magnesium concentration; (5) We found that only 29% of the children exceeded the RDI for magnesium intake, which further decreased with age; and (6) Magnesium intake was negatively correlated with the use of magnesium supplements.

In this study, we found that children treated with CNI showed significantly lower serum magnesium levels and that they were significantly more likely to develop hypomagnesemia. This is in line with previous findings in children [31,32,33,34] and adults [21]. In adults and animal models, it was shown that hypomagnesemia is caused by renal magnesium wasting [20,21,35]. CNI treatment lead to a decreased renal EGF production, resulting in a decreased magnesium channel TRPM6 expression in the distal convoluted tubule and, thus, a decreased renal magnesium reabsorption [20,35]. However, in children the pathogenesis of CNI induced hypomagnesemia is less clear as few studies have found a normal FE Mg^2+^ after CNI treatment, despite the development of hypomagnesemia [31,36]. In the present study, we demonstrated for the first time that the FE Mg^2+^ increased in children who developed hypomagnesemia when treated with CNI. This effect was independent from the magnesium intake as children who developed hypomagnesemia had an equal magnesium intake compared to normomagnesemic children. In addition to this finding, the urinary EGF concentration and the FE Mg^2+^ were inversely correlated, thus supporting the role of EGF in the renal magnesium reabsorption in children. In a multivariate model, EGF remained a significant predictor of the FE Mg^2+^. Our results pinpoint to a similar mechanism of renal magnesium reabsorption in children as was demonstrated in adults and rats: Renal EGF stimulates the magnesium reabsorption via the TRPM6 channel in the distal convoluted tubule. To strengthen this hypothesis, a study should be conducted investigating EGF and TRPM6 in renal biopsies. However, as this would indicate an invasive procedure in children, ethical approval can never be obtained in a study context alone. 

Additionally, from the multivariate model, age also appeared to be an independent predictor of the FE Mg^2+^ in children, with a positive correlation coefficient. In healthy children, it was shown that the FE Mg^2+^ did not correlate with age [37]. In the present study, only children with an underlying kidney disease were included. Several kidney diseases are accompanied by renal magnesium wasting in children, such as genetic disorders [38] or tubular dysfunction after acute tubular necrosis, or post-obstructive diuresis [39,40]. From our data, the FE Mg^2+^ increases with age in this population, which might indicate that the duration of the kidney disease is of importance in the development of renal magnesium wasting. As we did not go into detail on this finding, this would be an interesting topic for further research. 

In children, the renal function also predicts the FE Mg^2+^. A few other studies have already established a relation between the FE Mg^2+^ and the renal function. In children who recovered from ischemic acute tubular necrosis, it was shown that the FE Mg^2+^ declined, while the renal function improved [41]. This accords with the physiological principles as the FE Mg^2+^ is calculated from the serum creatinine (which is higher in patients with renal failure) and the urinary creatinine (which is lower in patients with renal failure). Therefore, an improvement of creatinine clearance would mathematically result in a decrease in the FE Mg^2+^. To overcome this mathematical issue, biomarkers of kidney function other than creatinine should be determined, such as urine neutrophil gelatinase-associated lipocalin (NGAL), chromium-51 EDTA, or cystatin-C, which have been shown to be reliable markers of acute kidney injury as well as chronic kidney disease [42,43,44]. In the present study, the renal function of the children with CKD (the CKD group) was also determined with chromium-51 EDTA analysis (data not shown), which showed a significant relation with the FE Mg^2+^. Also in pediatric patients after kidney transplantation, FE Mg^2+^ was negatively correlated with the renal function [45,46]. In adult patients, the renal function did not predict FE Mg^2+^ [21]. In that study, both the renal transplant group and the CKD control group were matched for renal function, which might explain why the relation was not found. In the present study, children with a decreased renal function (renal transplant group and CKD group), as well as children with a normal creatinine clearance (both nephrotic syndrome groups), were included. 

The protein/creatinine ratio was increased in all groups in this study population, however, below the nephrotic range proteinuria. Normal protein/creatinine ratios up to 340 mg/g were reported in healthy children [47], while nephrotic range proteinuria is defined as protein/creatinine ratio >2 g/g creatinine [48]. The protein/creatinine ratio did not differ between patients with and without hypomagnesemia and was not independently related to the FE Mg^2+^, suggesting that renal magnesium loss is not due to proteinuria.

Only 29% of the children reached the recommended amount of magnesium intake, which negatively correlated with age, with a higher magnesium intake more likely at a younger age. A few other European studies also found that older children had a higher prevalence of inadequate magnesium intake compared to younger children [8,49,50]. The average magnesium intake was the highest in the youngest children (<8 year), while the lowest intake was in the 8 to 12 year old children. From then on, the average magnesium intake increased with age which was also found in one other report [50]. In our study cohort, this was explained by the use of magnesium supplements, a treatment that is prescribed to patients with hypomagnesemia or patients susceptible to the development of hypomagnesemia. Our findings contrasted with a study of white and African-American girls, in which the magnesium intake decreased with age [51].

Our study has several strengths that led to new insights into the pathogenesis of CNI-induced magnesium loss in children. Despite the low incidence of nephrotic syndrome and renal transplantation in children, we were able to include sufficient numbers of patients. The longitudinal study design, with a low drop-out rate and age, gender, and renal function, matched the control groups and enabled us to draw sound conclusions. The most important limitation of the present study is that the CNI treated patients were included when they were already on treatment with CNI, sometimes for several years already. It would be of interest to include the patients at the moment CNIs are initiated and perform a long-term follow-up study over several years to study the time course influence of CNI on the renal EGF expression and renal magnesium loss. However, as CNI treatment is rare in children, with the low incidence of CNI-dependent nephrotic syndrome and kidney transplantation, the inclusion of sufficient children would take years.

## 5. Conclusions

In CNI-treated children who developed hypomagnesemia, the FE Mg^2+^ was increased. The urinary EGF concentration was an independent predictor of the FE Mg^2^, as were age and renal function.

## Figures and Tables

**Table 1:** Demographic data of the study groups.

Group	Renal Tx + CNI (*n* = 23)	CKD − CNI (*n* = 24)	NS + CNI (*n* = 27)	NS − CNI (*n* = 22)
Age (year)	13.4 (2.2–20.3)	11.1 (3.2–18.9)	12.5 (3.1–19.5)	12.3 (3.7–18.7)
Gender (M/F; %)	87/13	62/38	71/29	64/36
Length (cm)	151 (88–183)	145 (95–176)	149 (92–190)	149 (101–183)
Weight (kg)	43 ± 17	42 ± 16	46 ± 23	42 ± 15
BMI *z*-score	0.40 (−2.10–1.13)	0.24 (−2.24–2.05)	0.17 (−1.57–2.12)	−0.09 (−1.86–2.82)
Mg^2+^ intake (% of RDI)	89 (37–684)	86 (63–436)	87 (45–299)	86 (41–383)
Patients who exceeded the RDI for Mg^2+^ intake (%)	27.3	30.4	30.8	42.1
Mg^2+^ supplements (%)	13.0	8.0	13.8	25.0

Normally distributed variables are presented as mean ± SD; skewed data are presented as median (minimum-maximum). None of the presented variables significantly differed between the groups. Tx: Transplantation; CNI: Calcineurin inhibitor, CKD: Chronic kidney disease, NS: Nephrotic syndrome, RDI: Reference daily intake (corrected for age).

**Table 2:** Urine and serum analyses; data are presented in 4 groups.

Group	Renal Tx + CNI (*n* = 23)	CKD − CNI (*n* = 24)	NS + CNI (*n* = 28)	NS − CNI (*n* = 22)
Serum Creatinine (mg/dL)	1.11 (0.08) §#	1.27 (0.14) #§	0.72 (0.07) #$*	0.54 (0.03) $*§
Creatinine clearance (mL/min/1.73 m^2^)	59 (3) §#	62 (6) #§	98 (5) #$*	117 (4) $*§
Urinary protein/creatinine (mg/g)	347.4 (71.8) $	613.2 (115.0) *	848.0 (333.9)	544.9 (215.8)
Serum Mg^2+^ (mg/dL)	0.76 (0.02) #$	0.82 (0.02) *§	0.78 (0.02) #$	0.84 (0.01) *§
HypoMg (%)	39.1 $#	16.0 *§	44.8 $#	10.0 *§
FE Mg^2+^ (%)	7.82 (0.84 ) §#	7.76 (0.84)	3.95 (0.32) *$	3.57 (0.28) *$
CsA levels (ng/mL)	666 (45)	-	579 (38)	-
Tacrolimus levels (ng/mL)	8.62 (0.91)	-	7.71 (0.79)	-
Serum EGF (pg/mL)	776.6 (39.9)	742.1 (47.8) §	865.5 (40.7) $	817.2 (55.0)
Urine EGF (ng/mL)	7.0 (1.1) #§	11.5 (2.4) #§	35.4 (6.0) $*$	47.7 (6.6) *$
Urine EGF/creatinine (ng/mg)	0.11 (0.01) $#§	0.19 (0.03) #*§	0.33 (0.05) #$*	0.51 (0.07) $*§

The renal Tx patients treated with CNI, the CKD patients, the nephrotic syndrome patients treated with CNI, and the nephrotic syndrome patients not treated with CNI. Data were analysed with GEE and, therefore, presented as mean (SE). Tx: Transplantation, CNI: Calcineurin inhibitor, CKD: Chronic kidney disease, NS: Nephrotic syndrome, CsA: Cyclosporine, EGF: Epidermal growth factor, FE: Fractional excretion. # *p* < 0.05 vs NS-CNI; $ *p* < 0.05 vs CKD-CNI; * *p* < 0.05 vs Renal Tx + CNI; § *p* < 0.05 vs NS+CNI.

**Table 3:** Comparison of clinical and laboratory data between patients who developed hypomagnesemia and nomomagnesemic patients.

Group	Normomagnesemic Patients (*n* = 69)	Hypomagnesemic Patients (*n* = 28)	*p*-Value
Age (year)	11.79 (3.20–18.66)	13.91 (3.14–19.53)	0.492
Length (cm)	147 (94.5–190)	155.4 (92–77.5)	0.582
Weight (kg)	42.95 ± 18.39	43.61 ± 19.31	0.683
BMI *z*-score	0.14 (−2.24–2.82)	0.13 (−1.66–2.12)	0.418
Mg^2+^ intake (% of RDI)	87 (41–436)	88 (37–684)	0.692
Patients who exceeded the RDI for Mg^2+^ intake (%)	31.3	34.6	0.419
Serum Creatinine (mg/dL)	0.88 (0.06)	1.01 (0.11)	0.287
Creatinine clearance (mL/min/1.73 m^2^)	86 (4)	75 (6)	0.122
Urinary protein/creatinine (mg/g)	625.2 (155.8)	561.7 (120.5)	0.747
Serum estradiol (pg/mL)	60.28 (15.83)	67.24 (37.75)	0.865
Serum Mg^2+^ (mg/dL)	0.83 (0.01)	0.70 (0.01)	<0.001
FE Mg^2+^ (%)	5.41 (0.43)	6.55 (0.72)	0.178
CsA levels (ng/mL)	566.13 (30.70)	685.75 (53.56)	0.053
Tacrolimus levels (ng/mL)	6.90 (0.64)	8.55 (0.99)	0.163
Serum EGF (pg/mL)	802.77 (27.93)	795.60 (40.08)	0.883
Urine EGF (ng/mL)	27.46 (3.50)	18.58 (4.52)	0.120
Urine EGF/creatinine (ng/mg)	0.31 (0.03)	0.22 (0.05)	0.120

Cross-sectional data were analysed with Mann-Whitney-U test and presented as the median (minimum-maximum) or Student’s t-test (mean ± SD). Longitudinal data were analysed with GEE and, therefore, presented as the mean (SE). RDI: Reference daily intake, EGF: Epidermal growth factor, FE: Fractional excretion, CsA: cyclosporine.

**Table 4:** Log EGF as a predictor of FE Mg^2+^.

	β	*p*-Value	95% CI	
			Lower	Upper
Log Urinary EGF (ng/mL)	−2.084	<0.001	−3.153	−1.015
eGFR (mL/min/1.73 m^2^)	−0.049	<0.001	−0.067	−0.032
Serum Mg^2+^ (mg/dL)	−6.239	0.034	−12.014	−0.463
Age (year)	−0.239	0.001	−0.384	−0.094
Constant	20.078	<0.001		

In this population, the FE Mg^2+^ can be calculated using the following formula: FE Mg^2+^ = 20.078 − 2.084 × log Urinary EGF − 0.049 × eGFR − 0.239 × Age − 6.239 × serum Mg^2+^. eGFR: estimated glomerular filtration rate, EGF: epidermal growth factor.
